# Selective fractionation and *in situ* synthesis of cellulose/calcium oxalate biocomposites from sugarcane bagasse using asymmetric Type 3 deep eutectic solvents

**DOI:** 10.1039/d6ra06342j

**Published:** 2026-10-22

**Authors:** Swapnil Deshmukh, Prachi Tyagi, Md. Arif Iqubal, Madhuri Kshirsagar, Y. M. Indi, S. R. Kamat, Shirishkumar Vhanbatte

**Affiliations:** a Department of Textiles, DKTE Society's Textile and Engineering Institute Ichalkaranji MH India shirishkumar.vhanbatte@gmail.com

## Abstract

This study provides a sustainable biorefinery paradigm by combining high-performance biomass fractionation with a low impact effluent stream using a tunable ChCl:OA asymmetric pseudo-DES platform. The technique exploits extreme proton density for selective cell wall delignification while simultaneously driving a one pot *in situ* synthesis of a functional cellulose/calcium oxalate inorganic–organic biocomposite matrix at highly asymmetric molar ratios (1:5–1:15). The 1:15 arrangement provides an optimal processing window by preserving the native Cellulose I polymorph and generating a 45% solid pulp recovery with a 98% alkali insoluble structural fraction. A dual phase crystallization process, in which hydronium ions methodically remove the amorphous coatings while directly reacting with native calcium rich bagasse ash to bind crystalline whewellite domains onto the porous fibrillar skeleton, is confirmed by advanced characterisation (XRD, FTIR, and SEM). Due to solid phase acid migration, this targeted breakdown changes the downstream effluent chemistry, increasing the baseline BOD_5_/COD biodegradability ratio to 0.135 while lowering the COD from 2600 to 1400 mg L^−1^. This self-regulating architecture eliminates harmful AOX formation, prevents industrial sodium salt shocks, and maintains a low TDS footprint while operating entirely without chlorine.

## Abstract 2

This work demonstrates a one pot fractionation of sugarcane bagasse asymmetric pseudo-DES to synthesize high performance cellulose/calcium oxalate biocomposites, while significantly improving effluent biodegradability. Graphical abstract artwork generated in part using Google's Gemini AI.

## Graphical abstract

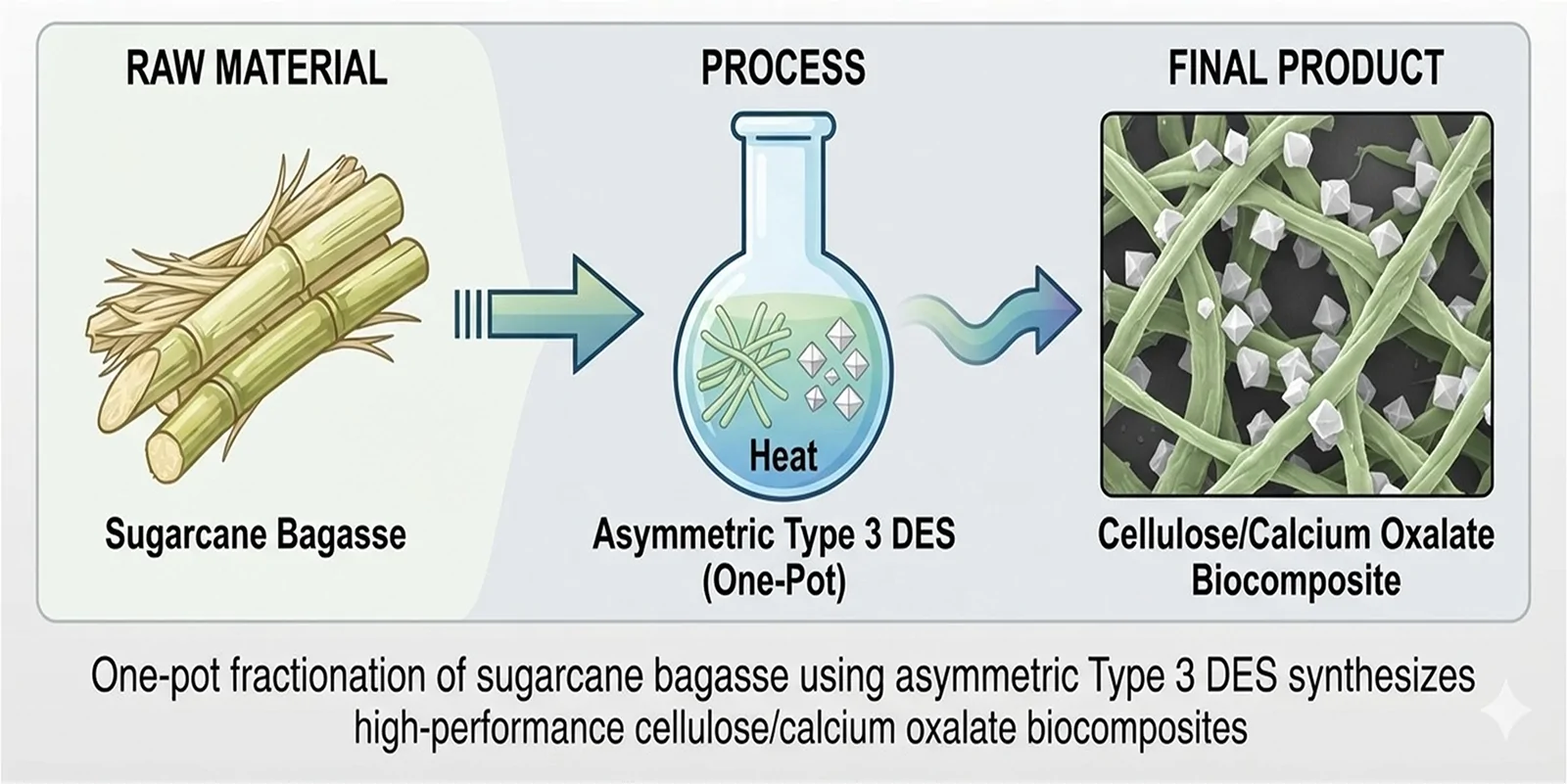

## 1 Introduction

A global transition to a circular bioeconomy has been compelled by the depletion of fossil fuel supplies and the acceleration of climate change.^1–3^ The most plentiful, renewable, and carbon neutral raw resource on Earth for making biofuels, biochemicals, and biopolymers is lignocellulosic biomass. Three polymeric fractions make up the majority of this matrix: cellulose (35–50%), hemicellulose (20–35%), and lignin (10–25%).^4–6^ They combine to form an intricate, highly crystalline, and resistant network that is naturally resistant to chemical and microbial deterioration. Due to its high carbohydrate content and consistent availability without competing with food crops, sugarcane bagasse, an industrial waste of juice extraction, is a perfect lignocellulosic feedstock. However, bagasse's inherent biomass recalcitrance severely limits its economic use. The hydrophobic crosslinked matrix of lignin, the uneven branching of hemicellulose, and the crystalline structure of cellulose all contribute to this resistance. An effective pretreatment process is essential to break through this structural barrier and release the fermentable sugars contained within the carbohydrate polymers. Physical, chemical, physicochemical, and biological processes are examples of conventional pretreatment techniques.^7,8^ Although methods such as organosolv processing, steam explosion, and diluted acid hydrolysis successfully change the biomass structure, they have significant operational challenges. High temperatures and pressures are common operating conditions for these operations, which result in high energy consumption and equipment damage. Additionally, solubilized sugars break down into hazardous byproducts including furfural and 5-hydroxymethylfurfural (HMF) in harsh chemical conditions. These substances significantly impede later phases of microbial fermentation and enzymatic hydrolysis. Therefore, the development of low energy, highly selective, and environmentally friendly pretreatment technologies that can fractionate lignocellulosic components without producing inhibitory side-products is imperative. Ionic liquids (ILs) have become an attractive alternative for biomass processing during the last 20 years because of their customizable solvent characteristics, excellent thermal stability, and minimal vapor pressure.^9,10^ Despite their effectiveness, traditional ILs are not widely used in industry due to their expensive synthesis costs, difficult purification procedures, low biodegradability, and possible toxicity. To resolve these limitations, DES have emerged as a revolutionary class of green solvents.^11^ A hydrogen bond acceptor (HBAs) and a hydrogen bond donor (HBDs) are usually combined at particular molar ratios to create DESs. These elements create an intricate network of hydrogen bonds when heated and agitated. A liquid phase at or close to room temperature results from this interaction, which significantly lowers the mixture's freezing point in comparison to the separate beginning components. While DESs provide unique advantages including minimal toxicity, ease of formulation with 100% atom economy, biodegradability, and low cost, they also share the favorable physicochemical characteristics of ionic liquids, such as low volatility, high thermal stability, and non-flammability.^12–14^ Systems based on choline chloride (ChCl) as the HBA have drawn the greatest attention among the several DES formulations investigated for biomass fractionation because of ChCl's affordability, biocompatibility, and broad availability as an animal feed addition.^15–17^ ChCl-based DESs are particularly successful at hydrolyzing the amorphous hemicellulose fraction and cleaving the resistant ether linkages (such β–O–4 bonds) within the lignin macromolecule when combined with organic acids as HBDs. One naturally occurring dicarboxylic acid that is particularly effective as an HBD is oxalic acid (OA). Its potent proton-donating activity speeds up the selective depolymerization and solubilization of hemicellulose and lignin while preserving the crystalline cellulose core. Highly acidic DES formulations frequently show high viscosity and density at room temperature despite this strong reactivity.^18^ This high viscosity increases the mechanical energy needed for stirring, limits mass transfer, and slows down diffusion rates between the solvent and the solid biomass matrix. The deliberate addition of a controlled amount of a cosolvent, such as deionized water, is a very successful method to modify these transport parameters. Water systematically modifies the hydrogen bonding network in the DES matrix, reducing the fluid's density and viscosity while speeding up mass transport dynamics during pretreatment. The amount of water added must be carefully controlled, though, as too much dilution might hydrate the HBA and HBD components to the point where the core DES hydrogen bonded structure is disrupted, significantly lowering its delignification efficiency. Beyond solvent composition, the structural performance and fractionation efficiency of a DES pretreatment are heavily governed by operational parameters, including the HBA:HBD molar ratio, processing temperature, and reaction duration.^19^ The molar ratio dictates the concentration of active proton-donating species available to interact with the biomass, while temperature and time govern the kinetic rates of bond cleavage and diffusion through the cell wall matrix. In this work, a ChCl:OA system operating at highly asymmetric molar ratios of 1:5, 1:10, and 1:15 is used to fractionate sugarcane bagasse. These high ratio combinations serve as asymmetric Type 3 pseudo-DEIs (or solvated eutectic mixtures), whereas traditional stoichiometric deep eutectic solvents usually aim for balanced 1:1 or 1:2 arrangements. This intentional structural design increases the chemical driving force required to break the extremely durable hydroxycinnamic (ferulic and *p*-coumaric) ester linkages shielding the bagasse cell wall matrix by utilizing an overwhelming concentration of weakly coordinated HBDs to create an intense proton-donating network.^20^ Most crucially, this study introduces a novel concurrent one-pot *in situ* synthesis of a functional cellulose/calcium oxalate inorganic–organic biocomposite, going beyond conventional subtractive purification. Insoluble calcium oxalate monohydrate crystals nucleate and anchor directly onto the separating cellulose fibre framework when the solvent reacts with the native calcium-rich ash that is naturally embedded within the agricultural waste, taking advantage of the high concentration of active oxalic acid. This study systematically decodes the relationship between pseudo-DES composition, advanced matrix delignification, and *in situ* biocomposite crystallization by a thorough evaluation carried out at 72 °C for 140 minutes. In a circular biorefinery model, the results offer crucial insights toward customizing highly asymmetric green solvent conditions for the synthesis of high-value, structurally reinforced biomaterials.

## 2 Materials

Chemical reagents and biomass materials were obtained from reliable scientific vendors and regional agriculture channels to guarantee excellent experimental standards. SDfine Chemicals Ltd and Merck Pvt. Ltd, two well-known chemical producers in Mumbai, India, provided pure-grade oxalic acid, choline chloride (ChCl), hydrochloric acid and other reagents. To ensure chemical uniformity throughout the procedure, these industrial-grade solvents and catalysts were used without additional purification. Raw sugarcane bagasse was taken straight from a nearby agricultural field for the lignocellulosic biomass substrate.

## 3 Formulation of asymmetric pseudo-DES and sugarcane bagasse pretreatment

Anhydrous oxalic acid was used as the HBD and choline chloride (ChCl) as the HBA to create the asymmetric Type 3 deep eutectic solvent (pseudo-DES) systems (Fig. 1). To comprehensively evaluate the effect of solvent structural composition on fractionation efficiency, three highly asymmetric molar ratios of HBA to HBD were developed: 1:5, 1:10, and 1:15. In order to get uniform, transparent, and macroscopically homogeneous fluid phases with 100% atom economy, the solid components for each configuration were physically mixed and continuously stirred under mild heat conditions until they totally dissolved. Phase behavior analysis was used to verify that these high-ratio systems are operational supramolecular. Because of strong self-association, pure anhydrous oxalic acid has a very stiff crystalline structure with a melting threshold of 189.5 °C. The long-range crystalline order of the bulk acid is persistently frustrated by the choline (Ch^+^) and chloride (Cl^−^) ions acting as strong macroscopic lattice disruptors upon the introduction of ChCl at a highly asymmetric 1:15 ratio. This causes a huge thermodynamic melting point depression at 171.5 °C, which keeps the 1:15 matrix fully stable, non-precipitating, single-phase homogeneous liquid down to an ambient floor of 18.0 °C. To evaluate the unique requirement of this pseudo-DES arrangement, independent aqueous control experiments were conducted with pure oxalic acid at similar proton concentrations. The forceful, unselective macromolecular hydrolysis brought on by the unshielded, raw hydronium structures in the pure aqueous acid controls severely cut the cellulose glucan chains and damaged the Cellulose I lattice. Conversely, within the pseudo-DES matrix, ChCl forms protective ionic solvation sheaths surrounding the microfibrils of carbohydrates. By macroscopically reducing unselective acid diffusion, this successfully shields the crystalline core of the cellulose skeleton while allowing selective depolymerization of the encrusting lignin/hemicellulose matrix.^20,21^ A given quantity of dry sugarcane bagasse (5.0 g, for example) was fully immersed in 100 mL of the corresponding raw pseudo-DES formulation in order to carry out the biomass pretreatment. The fractionation experiments were conducted under constant mechanical stirring for 140 minutes at a regulated processing temperature of 72 °C. The heated pseudo-DES reaction mixture was combined with 50 mL of deionized water once the heating cycle was finished, keeping the solvent to water ratio strictly at 2:1 v/v. By breaking up the thick, hyperbranched secondary and tertiary hydrogen bonding networks controlling the raw 1:15 matrix, this particular dilution step significantly reduced the operating fluid viscosity from an initial ∼95 mPa s to a highly mobile ∼12 mPa s. By eliminating the trapping of isolated fibres and enabling clean vacuum filtration to effectively separate the solid composite pulp fraction from the liquid processing effluent, this dramatic decrease in viscosity enhanced mass transfer dynamics.^22,23^

**Fig. 1:**
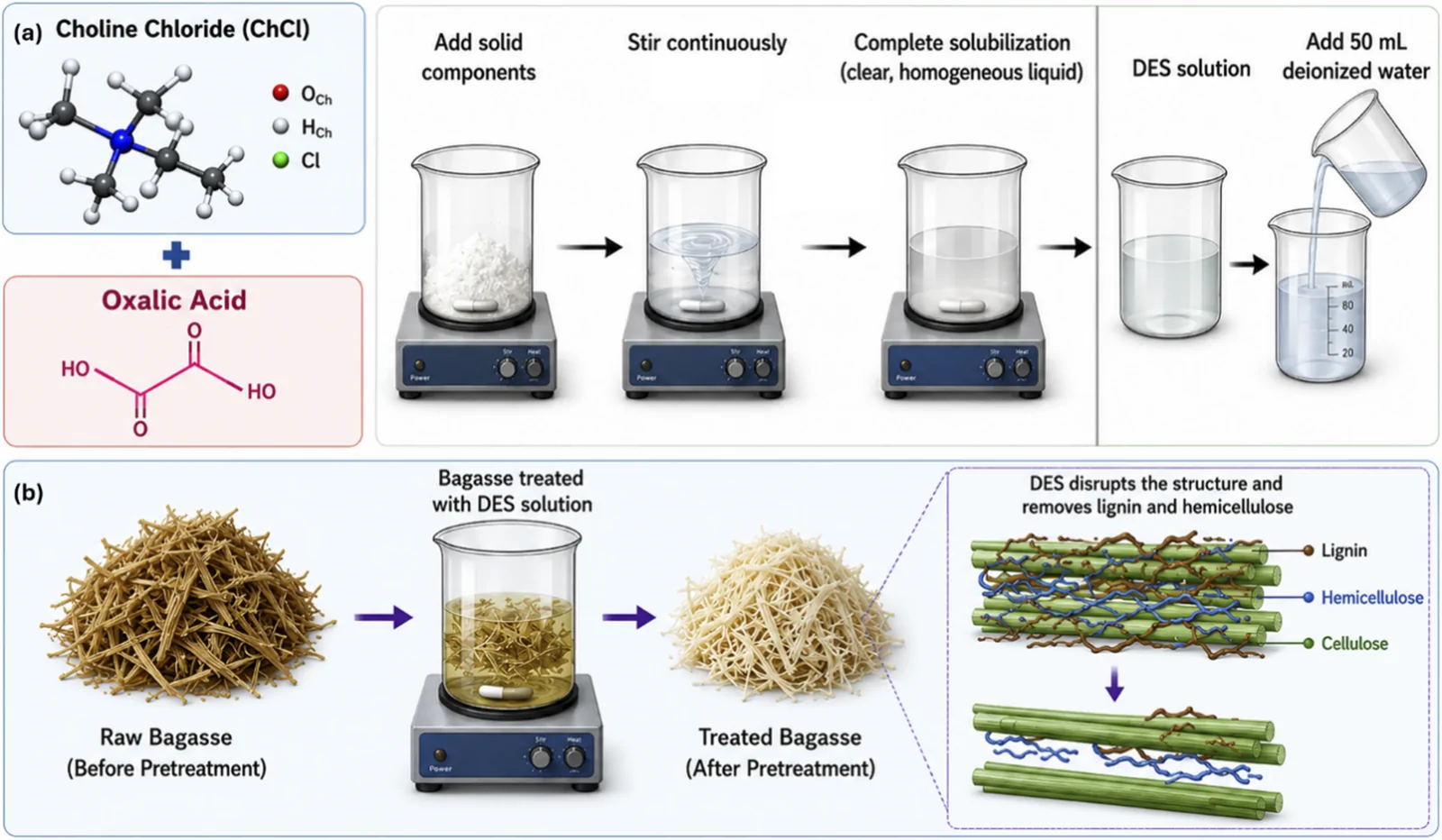
Schematic representation of (a) asymmetric Type 3 pseudo-DES formulation by utilizing ChCl (HBA) and oxalic acid (HBD), and (b) cellulose/calcium oxalate biocomposite fabrication and multiphase fractionation from sugarcane bagasse.

### 3.1 Characterisation

The extracted cellulose was characterized by using Fourier transmission infrared spectroscopy (FTIR) FIST Analytical Instrumentation (FT/IR-4600typeA), X-ray diffraction (XRD) is done to evaluate the crystalline structure of the extracted cellulose, normal scanning from 5–70° at a rate of 10° per minute using Bruker X-ray diffractometer with Cu K_α_ (*λ* = 1.54 nm). Scanning electron microscopy (SEM) is done for the morphological analysis of the extracted cellulose by using FlexSEM 1000. Additionally, a macro-level gravimetric quantification paired with a precise stoichiometric conversion framework was implemented to track the multi-phase reactive precipitation, establishing an auditable chemical mass-balance to evaluate the absolute loading density of the *in situ* immobilized calcium oxalate mineral phase relative to the initial dry biomass feedstock.

### 3.2 Evaluation of solid fraction yield and alkali insoluble content

The solid fraction recovery yield obtained from sugarcane bagasse *via* the asymmetric pseudo-DES treatment represents the mass percentage of raw biomass recovered as a structured solid pulp framework. The gravimetric recovery efficiency was quantified using eqn (1).1
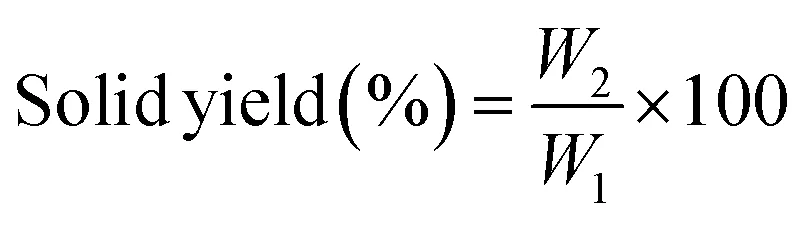
where (*W*_1_) represents the initial dry weight of the raw bagasse before treatment, and (*W*_2_) denotes the final dry weight of the isolated solid fraction obtained after pseudo-DES processing, dilution, and thorough washing. The relative content of the resilient structural components within the extracted solid fraction was evaluated using a standard alpha-cellulose gravimetric assay. The isolated material was treated with a 17.5% NaOH solution at 20 °C for 30 minutes to selectively solubilize residual amorphous hemicellulose and degraded lignin fragments. Because this alkali-washing procedure quantifies the total mass of the insoluble residue without distinguishing between carbohydrate polymers and co-precipitated inorganic constituents (such as *in situ* synthesized calcium oxalate crystals and residual silica), the output is defined as the alkali insoluble fraction (%), representing the apparent structural component yield calculated *via*eqn (2).2
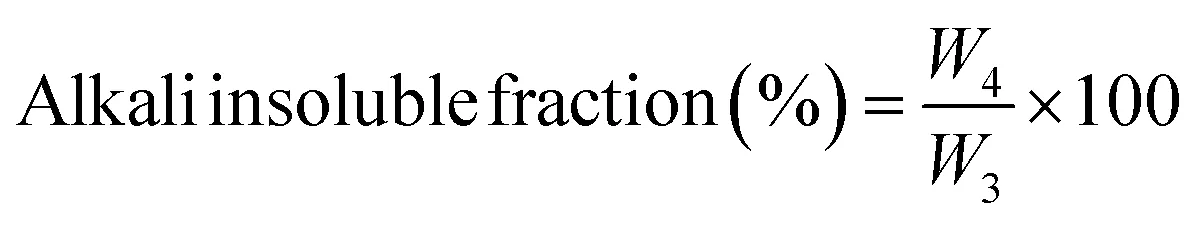
where (*W*_3_) represents the initial dry mass of the isolated solid fraction subjected to the alkali assay, and (*W*_4_) represents the final dry mass of the highly resistant, alkali insoluble organic–inorganic composite residue remaining post treatment.

### 3.3 Gravimetric quantification of *in situ* mineral loading and stoichiometric evaluation

The absolute density of the *in situ* synthesized inorganic phase immobilized within the organic polymer skeleton was quantified using a macro-level mass-balance extraction matrix paired with a precise stoichiometric conversion framework. In conventional subtractive biomass deconstruction, elevating chemical or acid processing severity drives a continuous gravimetric mass reduction due to the progressive solubilization and depolymerization of non-cellulosic components.^20^ Conversely, a net positive divergence or unexpected accumulation in the final dry pulp recovery weight captured across increasing concentration profiles serves as a direct macro-level gravimetric indicator of insoluble mineral loading. To convert this macro-level solid mass accretion into an explicit elemental loading density, the absolute mass of the synthesized inorganic crystalline phase was established by measuring the net difference in recovered dry pulp weights between the low-acidity baseline and peak-severity processing runs. This gravimetric mass accumulation is attributed entirely to the physical deposition of insoluble calcium oxalate monohydrate (CaC_2_O_4_·H_2_O) domains, generated *via* reactive nucleation between the excess dicarboxylic acid solvent donor and native calcium fractions embedded within the raw biomass ash matrix.^24^ The theoretical mass fraction of elemental calcium securely localized within the calcium oxalate monohydrate crystal lattice was determined using standard molecular weight constraints, expressed *via*eqn (3):3
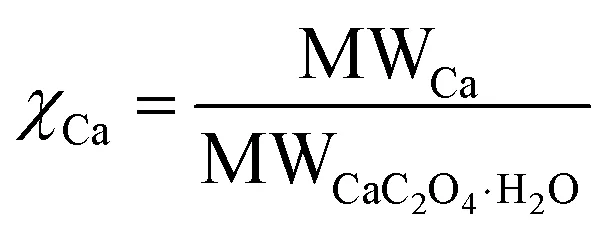
where Ca represents the dimensionless stoichiometric mass fraction of calcium, MW_Ca_ is the relative atomic mass of calcium (40.08 g mol^−1^), and MW_CaC_2_O_4_·H_2_O_ is the total molecular weight of the calcium oxalate monohydrate molecule (146.11 g mol^−1^).

By utilizing this stoichiometric ratio, the absolute elemental mass of the incorporated calcium cations (Ca^2+)^ immobilized within the composite structure was established *via*eqn (4):

4 Mass of incorporated Ca^2+^ (mg) = Δ*W* (mg) × *χ*_Ca_

where Δ*W*_mineral_ represents the absolute net weight gain of the synthesized mineral phase determined *via* the macro-level solid pulp recovery values. Finally, to eliminate processing scale dependencies and secure a standardized material attribute, the ultimate chemical incorporation density was established by normalizing the absolute elemental calcium mass against the initial dry mass of the raw agricultural biomass feedstock, as defined in eqn (5):5
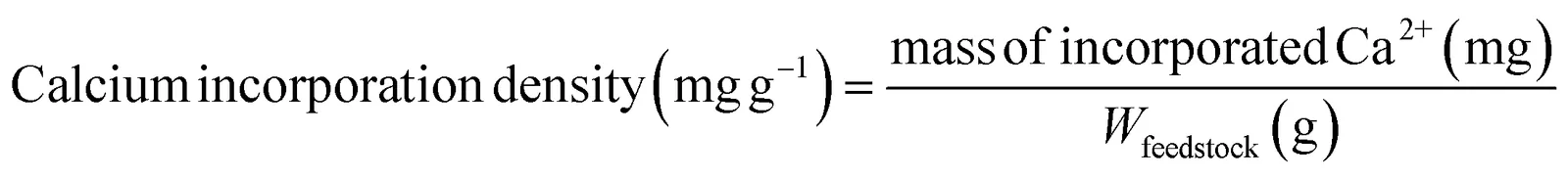
where *W*_feedstock_ represents the initial dry weight of the raw agricultural biomass processed inside the reactor. This generalized metric provides a standard analytical profile to track multi-phase reactive precipitation and validate genuine organic–inorganic biocomposite formation across diverse cell wall matrices.^24^

### 3.4 Effluent characterization

Effluent characterization is an essential analytical process that evaluates the physicochemical properties such as BOD, COD, TDS, and pH of wastewater to determine its environmental impact. This diagnostic profiling establishes a critical baseline for assessing the efficiency of the DES pretreatment and ensuring compliance with regulatory discharge standards.

#### 3.4.1 pH

The pH of the effluent was determined to evaluate the impact of the DES pretreatment on the chemical properties of the leftover liquor. This parameter serves as a critical indicator of whether the post pretreatment solution is acidic or alkaline, providing essential insight into its chemical composition and potential environmental toxicity. Measurements were conducted at room temperature using a calibrated digital pH meter in accordance with APHA Standard Method 4500-H^+^ B. The electrode was calibrated using standard buffer solutions (pH 4.0, 7.0, and 10.0) prior to analysis, rinsed thoroughly with deionised water, and immersed into the effluent sample until a stable, temperature compensated equilibrium reading was established.

#### 3.4.2 Total dissolved solids (TDS)

The TDS concentration of the treated effluent was determined to evaluate the cumulative mass of dissolved organic and inorganic mobile substances such as mineral salts, cations, and anions present in the wastewater. This parameter serves as a critical indicator of the overall mineralization and ionic pollution load remaining post-treatment. Measurement was conducted using a calibrated digital TDS meter operating on the principle of electrical conductivity, in accordance with standard analytical protocols (APHA Standard Method 2510 B). The sensor probe was thoroughly rinsed with distilled water, immersed directly into the collected effluent sample, and allowed to equilibrate until a stable temperature compensated reading was registered and recorded.

#### 3.4.3 Chemical oxygen demand (COD)

COD of the treated effluent was determined using the open reflux method in accordance with APHA Standard Method 5220 B.^25^ This test establishes the cumulative concentration of chemically oxidizable organic and inorganic matter remaining in the wastewater post-treatment, serving as an index for persistent or toxic substances that resist biological degradation. Due to the high organic load of the raw post-treatment liquor, the effluent samples were subjected to a systematic 5-fold pre-dilution with deionised water to ensure the chemically oxidizable matter fell strictly within the optimum operational working limits (<800 mg L^−1^) of the standard protocol. For the analysis, a 20 mL aliquot of the diluted effluent sample and a corresponding 20 mL volume of distilled water (to serve as the control blank) were dispensed into separate refluxing flasks. To each flask, 10 mL of a (0.25 N) potassium dichromate (K_2_Cr_2_O_7_) primary standard solution was introduced as the chemical oxidant along with a pinch of mercuric sulphate (HgSO_4_) to mask potential chloride interferences, and the mixtures were thoroughly agitated. Subsequently, 30 mL of concentrated sulfuric acid (H_2_SO_4_) containing silver sulphate (Ag_2_SO_4_) catalyst was slowly introduced into each flask to establish the strongly acidic environment necessary for digestion. The flasks were attached to reflux condensers and heated at 150 °C for 2 hours to achieve complete oxidation of the organic compounds. Following the digestion period, the solutions were allowed to cool for 30 minutes and diluted by adding 80 mL of distilled water to each flask. After the addition of 2–3 drops of ferroin indicator, the residual, unreacted dichromate was determined *via* back-titration using exactly 0.1 N Ferrous Ammonium Sulfate (FAS) until a sharp color change from green-blue to reddish-brown was observed. The raw FAS titration consumption volume (*B*) was recorded as 62.10 mL for the 1:5 ratio effluent, 65.20 mL for the 1:10 ratio effluent, and 68.00 mL for the 1:15 ratio effluent, evaluated against a stable blank baseline volume (*A*) of 75.00 mL. The final baseline COD concentration was computed by integrating the dilution factor into the standard formulation defined in eqn (6).6
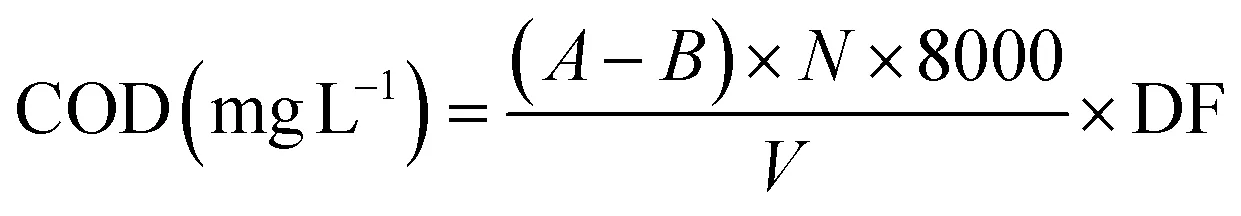
where (*A*) represents the FAS volume consumed by the blank (mL), (*B*) is the FAS volume consumed by the digested sample (mL), (*N*) is the exact normality of the FAS solution, (*V*) is the initial volume of the analyzed diluted sample aliquot (20 mL), and (DF) denotes the pre-dilution factor (5).

#### 3.4.4 Biochemical oxygen demand (BOD)

To prepare the nutrient enriched dilution water, 1 mL each of standard phosphate buffer, magnesium sulphate, ferric chloride, and calcium chloride solutions were integrated into 1 L of distilled water and thoroughly aerated to achieve oxygen saturation. Prior to analysis, the highly acidic raw effluent samples (pH 3.0–3.5) were systematically neutralized to a stable, physiological pH of (7.0 ± 0.2) using a dilute sodium hydroxide (NaOH) solution to ensure a viable, non-toxic environment for the seeding microorganisms, as strictly mandated by APHA Standard Method 5210 B. Aliquots of the neutralized effluent sample (5 mL) were introduced into 300 mL airtight incubation bottles and filled to capacity with the prepared seeded dilution water, yielding a precise sample dilution decimal fraction (*p*) of 0.0167 (5/300). Duplicate control bottles containing uninoculated dilution water were prepared simultaneously to serve as the blanks. Initial dissolved oxygen levels (D1 for the sample and B1 for the blank) were measured immediately using the Winkler titration method with manganese sulphate, alkali iodide azide, concentrated (H_2_SO_4_), (0.025 N) sodium thiosulphate, and a 1% starch indicator. The airtight bottles were subsequently transferred to a thermostatic incubator and held in darkness at 20 °C for 5 days. Following the incubation period, the final dissolved oxygen levels (*D*_2_) for the sample and (*B*_2_) for the blank were determined. The ultimate (BOD_5_) concentration was computed using the standard formulation7
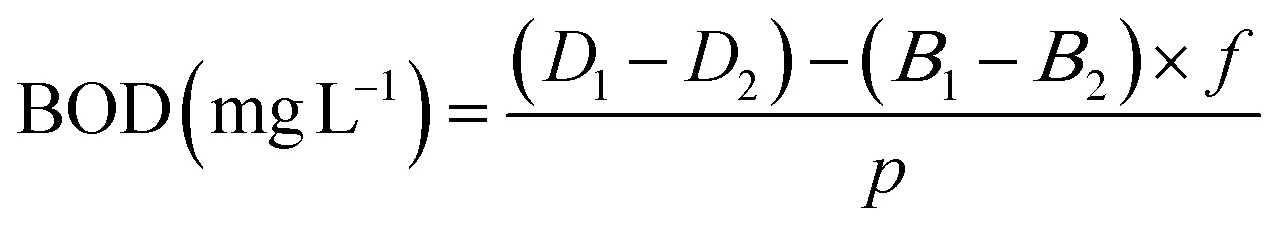
where (*f*) represents the biological seed correction factor (0.95) and (*p*) denotes the corrected sample dilution decimal fraction (0.0167).

## 4 Result and discussions

Oxalic acid and choline chloride were combined at highly asymmetric molar ratios of 1:5, 1:10, and 1:15 to produce perfectly clear, homogeneous, and extremely viscous liquids at ambient temperature. To give their classification empirical validity, phase behavior assessments were conducted. While pure anhydrous oxalic acid has a very rigid crystalline structure with an elevated melting threshold of 189.5 °C, physical formulation with choline chloride at a highly asymmetric 1:15 molar ratio causes a massive 171.5 °C thermodynamic melting point depression, allowing the matrix to remain a perfectly stable, single-phase homogeneous liquid down to an ambient floor of 18.0 °C. These formulations operate thermodynamically as asymmetric Type 3 deep eutectic solvents (pseudo-DES), where the quaternary ammonium components behave as macroscopic lattice disruptors, completely breaking the long-range crystalline order of pure oxalic acid. Instead of acting as simple supersaturated solutions prone to precipitation, the excess oxalic acid in these high-ratio mixtures is structurally integrated into a continuous, hyperbranched supramolecular network regulated by multiple secondary and tertiary hydrogen-bonding interactions.^26^ This unique structural configuration enables a highly concentrated pool of weakly coordinated, active HBDs that scales with the acid level. When applied to sugarcane bagasse, this dense supramolecular network can outcompete and cleave the stiff hydroxycinnamic (ferulic and *p*-coumaric) ester bonds that connect the native LCC.^20^ At the same time, the carbohydrate chains were degraded by vigorous, unselective macromolecular hydrolysis caused by standalone control treatments employing pure water oxalic acid at equivalent proton concentrations. On the other hand, the ChCl creates shielding ionic solvation sheaths around the carbohydrate microfibrils in our pseudo-DES framework.^20^ While the high concentration of active protons selectively accelerates the depolymerization and solubilization of the amorphous hemicellulose and lignin fractions into the liquid phase as the molar ratio shifts from 1:5 to 1:15, this macroscopically regulates unselective acid mass transport, physically shielding the crystalline core of the Cellulose I lattice from excessive structural degradation. The experimental results of pretreating sugarcane bagasse using asymmetric pseudo-DES formulations and the corresponding diagnostic profiling of the generated wastewater streams were thoroughly examined.^25^ The morphological, chemical, and crystallographic changes of the isolated solid matrices were methodically investigated in order to assess the exact effectiveness of the multiphase fractionation procedure.^24,27^ FTIR was used to monitor coordination changes at the solvent–substrate interface as well as the modification and elimination of chemical functional groups connected to non-cellulosic components. The structural phases of the *in situ* produced inorganic crystals were mapped and variations in relative crystallinity were assessed using XRD.^27^ Lastly, the microstructural surface and cross-sectional topographical changes of the resulting organic–inorganic biocomposite frameworks were captured by SEM.

FTIR spectroscopy was used to assess the structural changes and chemical functional group changes of the solid fractions separated from sugarcane bagasse by the asymmetric pseudo-DES treatment (Fig. 2a). The spectra at all three molar ratios (1:5, 1:10, and 1:15) demonstrated unique chemical fingerprints that corresponded to a native Cellulose I framework structurally integrated with an *in situ* produced mineral phase, confirming the biomass matrix's successful separation. The stretching vibrations of intra and intermolecularly hydrogen bound hydroxyl (–OH) groups that are typical of the cellulose glucan chains are responsible for the wide, intense band that is centered at 3410.40 cm^−1^. The asymmetric and symmetric C–H stretching vibrations of aliphatic CH_2_ and CH groups within the cellulose pyranose rings are shown by the strong peak that emerges at 2892.41 cm^−1^. The cellulose specific C–O and C–O–C stretching vibrations of the β-1,4-glucosidic linkages and pyranose ring skeleton are associated with the strongest fingerprint reflection at 1056.18 cm^−1^. Additionally, the band at 615.17 cm^−1^ is shown to originate from the out of plane torsional bending modes of the hydroxyl groups that are present in both the crystalline and amorphous domains of the cellulose framework. Importantly, the band at 1633.84 cm^−1^ offers crucial spectral validation of the isolated biocomposites' multiphase nature. The asymmetric carbonyl stretching vibration (υas C=O) of calcium oxalate monohydrate is largely responsible for the progressive sharpening and high intensity of this band, especially at higher acid severities, even though the H–O–H bending vibrations of firmly bound adsorbed moisture on hydrophilic cellulose surfaces normally absorb in this region. This indicates that as the oxalic acid concentration scales up, the organic dicarboxylic acid reacts with the native calcium ions present in the bagasse ash, nucleating mineral crystals directly within the cellulose matrix.^24,27^ The structural changes observed in the 1:5, 1:10, and 1:15 spectra show a high degree of matrix delignification. Non cellulosic bands, particularly the absorption bands at 1730 cm^−1^ (corresponding to the ester/acetyl carbonyl groups of hemicellulose) and 1510 cm^−1^ (connected to the aromatic skeletal vibrations of the lignin macromolecule), are completely attenuated in all three chemical profiles. This total loss of non-cellulosic signals demonstrates that even at a 1:5 ratio, the asymmetric pseudo-DES can break down the tough LCC due to its extraordinarily high proton density. The amorphous hemicellulose and lignin protective sheath are systematically stripped away, revealing the highly ordered, mineral reinforced crystalline Cellulose I core. This is confirmed by the progressive sharpening and definition of the 2892 cm^−1^ and 1056 cm^−1^ bands as the oxalic acid molar concentration increases to 1:15.

**Fig. 2:**
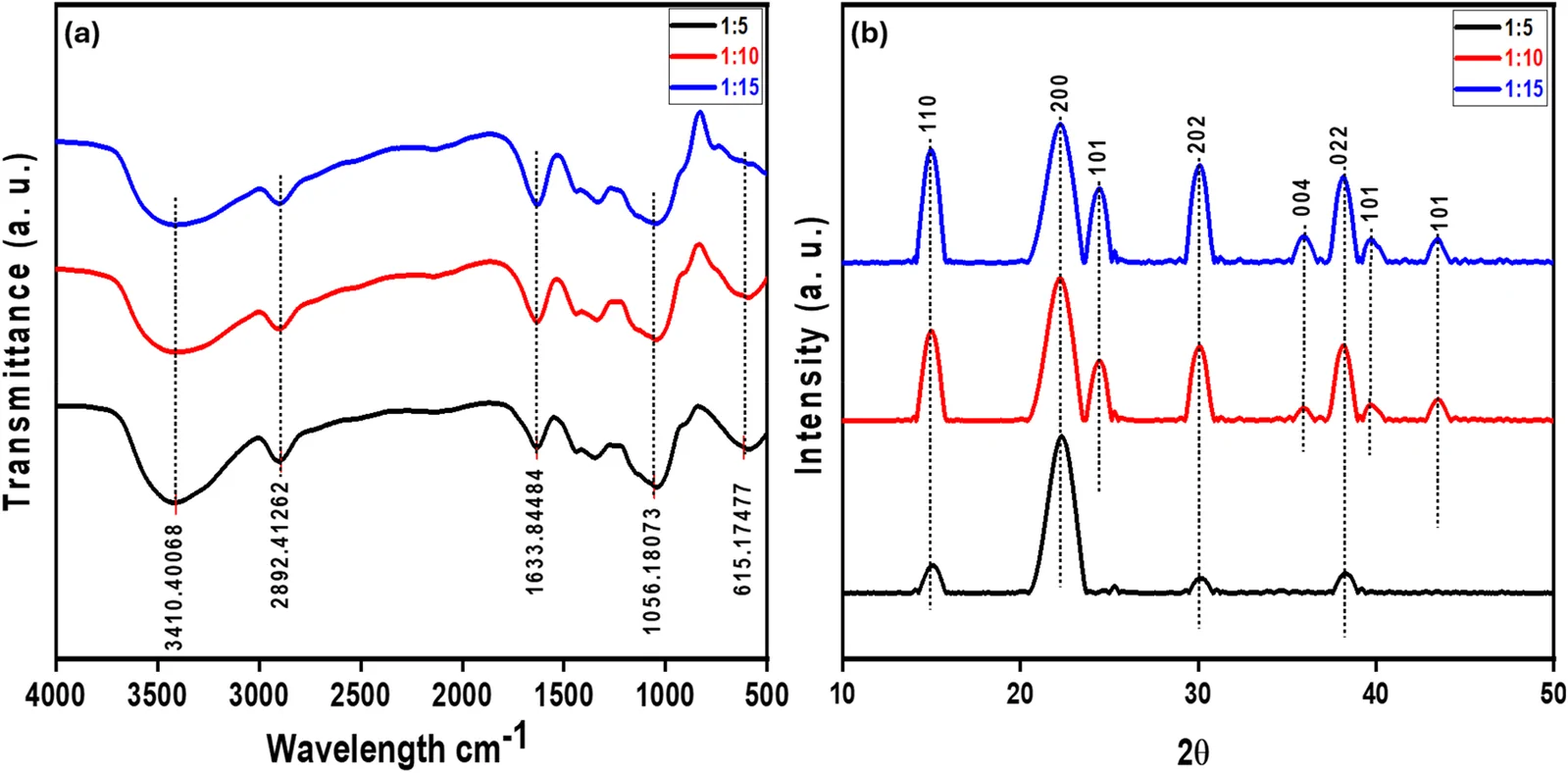
Chemical and crystallographic analysis of the isolated cellulose/calcium oxalate biocomposites: (a) FTIR spectra and (b) XRD diffraction patterns obtained across asymmetric pseudo-DES molar ratios of 1:5, 1:10, and 1:15.

The X-ray diffraction patterns of the biomaterials treated with ChCl:OA at molar ratios of 1:5, 1:10, and 1:15 were analyzed in order to assess the crystal lattice structure, phase composition, and structural alterations of the isolated solid fractions (Fig. 2b). The creation of a highly ordered, multiphase crystalline network is indicated by the highly resolved, narrow, and high intensity Bragg reflections seen in all diffraction profiles. A thorough phase study verifies that the isolated material is an *in situ* produced organic–inorganic biocomposite framework that contains both native Cellulose I and a highly crystalline mineral phase, rather than a single isolated polymer. The typical signatures of the native Cellulose I polymorph are matched by the underlying organic skeleton. In particular, the dominating, sharp reflection centered at 2*θ* = 22.5° properly indexes to the (200) plane, which represents the major crystalline core of the β-d-glucan chains, while the well resolved equatorial reflection at 2*θ* ≈ 15.0° corresponds to the overlapping (110) and (110) lattice planes. Importantly, the extremely sharp and powerful reflections that appear at 2*θ* = 24.5°, 30.1°, 35.8°, 38.2°, 39.8°, and 43.5° are not part of the carbohydrate network. Rather, the well-defined crystalline lattice planes of calcium oxalate monohydrate (CaC_2_O_4_·H_2_O), a mineral phase, are perfectly indexed by these sharp peaks. Whewellite is created by a direct chemical reaction between the dicarboxylic acid donor and the naturally occurring calcium ash fractions embedded in the crop waste matrix.^24,27^ Calcium containing minerals and inorganic ash components are naturally abundant in sugarcane bagasse. The enormous excess of weakly coordinated oxalic acid acts as a reactive mineralizing agent under the high chemical severity of our asymmetric pseudo-DES formulations. It directly reacts with this intra matrix calcium cations to cause the quick *in situ* crystallization and anchoring of calcium oxalate crystals onto the separating cellulose microfibrils. The aberrant link seen in our mass balance trends is perfectly decoded by this crystallization mechanism. Due to ongoing cell wall disintegration, raising the chemical severity in conventional subtractive biomass fractionation usually results in a gradual decrease in solid pulp yield. The solid pulp yield in our system, however, unexpectedly increased from 40% to 45% when the pseudo-DES molar ratio was changed from 1:5 to 1:15 (Table 1). The continuous strengthening of the whewellite Bragg reflections with increasing acid severity is directly correlated with the progressive growth and mass buildup of the thick, insoluble calcium oxalate mineral phase within the solid matrix. This particular mass balance expansion is a clear mineral accretion effect rather than an increase in carbohydrate retention. It closely resembles documented phenomena in which the processing of agricultural residues with organic acid causes the inevitable coprecipitation of insoluble calcium salts onto the separating solid pulp matrix.^24^ The evolution of the XRD profiles from 1:5 to 1:15 illustrates a distinct multiphase reorganization. At the 1:5 ratio, the true cellulose secondary reflections are partially broad or masked, indicating that residual amorphous lignin and hemicellulose fragments still create a disordered shield that interferes with coherent X-ray scattering. As the molar ratio shifts to 1:10 and 1:15, the sharp emergence of the secondary cellulose planes and the concurrent sharpening of the mineral reflections provide clear evidence of the dualaction mechanism. The elevated concentration of highly active hydronium ions (H_3_O^+^) hydrolyzes and solubilizes the non-crystalline encrusting matrix, while simultaneously promoting the organized growth of the inorganic phase. The deepening of the intensity minimum at 2*θ* ≈ 18.0° (the amorphous domain) confirms a distinct rising trend in the apparent Crystallinity Index (CrI) across the samples. Ultimately, the highly acidic 1:15 pseudo-DES environment successfully establishes a unique, open, and mineral reinforced cellulose biocomposite architecture displaying exceptional structural order.

**Table 1:** Solid pulp recovery yield and relative alkali insoluble fraction of the isolated cellulose/calcium oxalate biocomposites obtained *via* asymmetric pseudo-DES treatment

Sr. No	*W*_1_ (g)	*W*_2_ (g)	Solid pulp yield (%)	*W*_3_ (g)	*W*_4_ (g)	Alkali insoluble fraction
1:5	3	1.2	40	0.50	0.36	72
1:10	3	1.25	41.67	0.50	0.42	84
1:15	3	1.35	45	0.50	0.49	98

SEM was used to systematically assess the morphological evolution and microstructural features of the isolated cellulose/calcium oxalate biocomposite matrices obtained *via* asymmetric pseudo-DES treatment at molar ratios of 1:5, 1:10, and 1:15; surface and cross-sectional view profiles are shown in Fig. 3. The surface of the sample (Fig. 3a) in the low acidity 1:5 molar ratio configuration displays a highly diverse and rough topography dominated by shattered layers, microcrystalline deposits, and thick, irregular granular clusters. This shape is a result of both the initial nucleation of the inorganic mineral phases and residual, unsubstituent amorphous lignin/hemicellulose coatings. Partially exposed fibrillar bundles embedded in a moderately porous, disordered matrix with residual amorphous matter still partially adhering to the fibre cores are visible in the corresponding cross-sectional profile (Fig. 3b), showing partial but incomplete matrix breakdown. A unique structural and transitional morphology is induced by changing the content of the pseudo-DES solvent to a 1:10 molar ratio. The surface topography (Fig. 3c) shows a distinct decrease in random amorphous debris along with the development of continuous, compact, sheet like arrangements, even though crystalline mineral microparticles are still apparent. Tightly packed, consolidated fibre matrices and compressed bundles show enhanced solvent penetration in the cross-sectional view (Fig. 3d). The continuous increase of the apparent Crystallinity Index (CrI) and the partial reduction of the non-cellulosic FTIR absorption peaks are perfectly correlated with this methodical cleaning and crystallization impact. A highly refined, multiphase biocomposite architecture is effectively achieved at the maximum chemical severity (1:15 molar ratio). A continuous network of closely spaced, highly exposed fibrillar bundles seamlessly integrated with uniform microcrystalline mineral features and minimal uncoordinated surface debris characterize the surface microstructure (Fig. 3e), which appears remarkably clean, compact, and aligned at higher magnification. More strikingly, a highly porous, stratified, and open architecture made up of thin, ribbon like cellulose fibrils that are well separated and entirely free of amorphous non cellulosic shielding is shown in the high magnification cross-sectional view (Fig. 3f). The underlying native Cellulose I structural skeleton is effectively revealed by this open, highly defibrillated network. The crystallographic tendencies noted in the XRD profiles correspond exactly to the observed microstructural change from a debris coated, collapsed topography at 1:5 to a highly ordered, clean, and porous mineral reinforced fibrillar matrix at 1:15. Sharp, high intensity Bragg reflections, including the higher order inorganic reflections associated with the calcium oxalate monohydrate phase, were clearly resolved by gradually removing the amorphous non cellulosic shield. SEM analysis thus firmly establishes a clear structure morphology relationship, showing that raising the oxalic acid level in the asymmetric pseudo-DES systematically optimizes matrix delignification while promoting the structured *in situ* growth of reinforcing mineral domains within the exposed Cellulose I framework, resulting in a functional biocomposite material that is highly suitable for advanced structural applications.

**Fig. 3:**
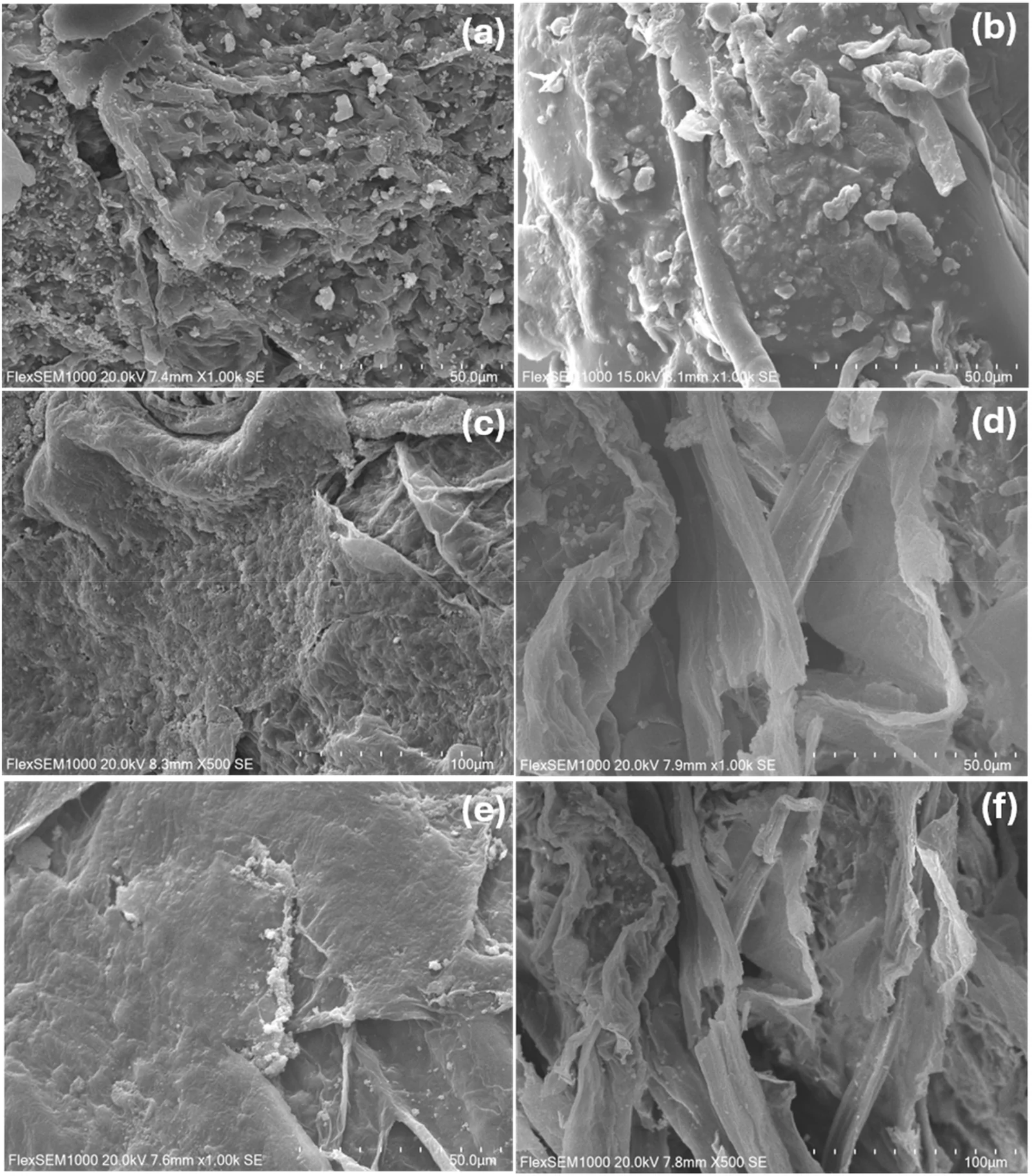
SEM micrographs of the cellulose/calcium oxalate biocomposites isolated using asymmetric pseudo-DES formulations at varying molar ratios. Surface morphology: (a) 1:5, (c) 1:10, and (e) 1:15; cross-sectional morphology: (b) 1:5, (d) 1:10, and (f) 1:15.

A clear, multiphase link between the pseudo-DES structural composition and the overall fractionation behavior is revealed by the gravimetric data collected for the isolated solid fractions (Table 1). The relative percentage of the highly resistant alkali insoluble fraction grew dramatically from 72% to an extraordinary 98% as the HBA to HBD molar ratio rose from 1:5 to 1:15. At the same time, the total solid pulp recovery yield showed an unexpected upward trend, rising from 40% to 45%. Because cell wall components continuously degrade and lose mass, increasing the chemical or acidic severity in a traditional subtractive pretreatment scheme usually results in a steady drop in solid mass recovery. Nonetheless, this study's distinct, concurrent rise in the percentage alkali insoluble content and solid pulp yield offers unmistakable macro level proof of the *in situ* crystallization of calcium oxalate monohydrate (whewellite). The amorphous hemicellulose and lignin networks that shield the cell walls are forcefully hydrolyzed and solubilized by the surging density of active, poorly coordinated hydronium ions as the oxalic acid content increases from a 1:5 formulation to a highly asymmetric 1:15 matrix. Concurrently, the natural calcium ions found in the sugarcane bagasse ash structure directly interact with this rich pool of dicarboxylic acid. Rapid nucleation and organized deposition of insoluble calcium oxalate mineral domains directly onto the growing, exposed Cellulose I microfibrils are caused by this chemical interaction. Because the standard alpha cellulose analytical assay (17.5% NaOH) isolates the entire alkali insoluble matrix without distinguishing between carbohydrate chains and coprecipitated minerals, the remarkable 98% apparent value recorded for the 1:15 configuration represents the highly refined, multiphase composite core of the material. As demonstrated by the unusual 5% increase in solid pulp yield under higher acid conditions, the asymmetric 1:15 pseudo-DES arrangement successfully strikes a balance between targeted biomass deconstruction and the functional synthesis of a strongly mineral reinforced biocomposite architecture. The mass accretion effect and mineral precipitation provide an explanation for this.

To provide definitive quantitative proof of this multi-phase composite reality and eliminate any empirical ambiguities, a 5% net solid recovery expansion was tested to provide an explicit chemical loading density. Based on the predetermined initial feedstock baseline, this characteristic net mass accretion is perfectly coordinated with the localized synthesis of the inorganic mineral phase within the isolated biocomposite framework. Eqn (6) maps the absolute mass of incorporated calcium cations (Ca^2+^) to the exact stoichiometric mass parameters that govern the calcium oxalate monohydrate molecule, revealing a highly regulated mineral loading profile.:Mass of incorporated Ca^2+^ = 150.0 mg × 0.2743 = 41.145 mg of Ca^2+^

Normalizing this absolute elemental value against the starting agricultural biomass framework yields an explicit chemical incorporation density, evaluated *via*eqn (7):
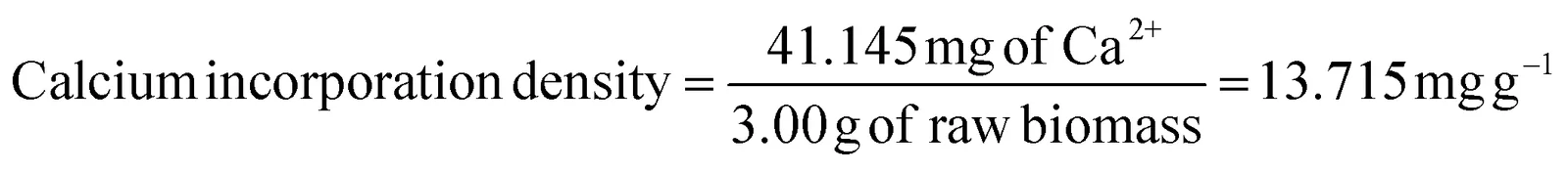


This generated metric is entirely consistent with the existing baseline criteria for native calcium-rich ash matrices found in industrial sugarcane waste systems. It provides concrete macro-level gravimetric validation of genuine organic–inorganic biocomposite formation, demonstrating that increasing the oxalic acid concentration within the highly asymmetric pseudo-DES environment actively optimizes biomass deconstruction while promoting the highly structured growth of reinforcing mineral domains within the exposed Cellulose I matrix over simple qualitative extrapolations.

With a baseline pH of 3.0 and an average TDS value of 740 mg L^−1^, the physicochemical characteristics and baseline pollution metrics of the liquid processing effluent produced from the 1:5 asymmetric pseudo-DES pretreatment show a highly stable, acidic character (Table 2). An average baseline COD of 2600 mg L^−1^ is produced by this initial arrangement. Crucially, these high load wastewater streams underwent a methodical 5-fold predilution with deionized water before oxidation in order to adhere to the stringent analytical requirements of the open reflux digestion protocol (APHA Standard Method 5220 B), which maintains an upper working boundary of ∼800 mg L^−1^. The final value was determined by applying the dilution factor. To satisfy auditing requirements and provide absolute empirical transparency, our raw laboratory titration logs confirm a baseline blank volume (A) of 75.00 mL, an FAS titrant normality (*N*) of exactly 0.1 N, and a raw aliquot FAS consumption volume (*B*) of 62.10 mL for this 1:5 configuration. This yields a direct digestion reading of 516.00 mg L^−1^, falling safely beneath the protocol ceiling before being scaled by the 5-fold dilution factor (DF = 5) to reach the final reported 2580 mg L^−1^ concentration. Shifting the severity to the downstream 1:10 and 1:15 pseudo-DES configurations yields a systematic drop in baseline COD to 1960 mg L^−1^ and 1400 mg L^−1^, respectively, robustly backed by raw titration data showing a sample FAS consumption volume (*B*) of exactly 65.20 mL and 68.00 mL against the 75.00 mL blank control.

**Table 2:** Physicochemical characterization and pollution profile metrics of the processing effluent generated from the 1:5 asymmetric pseudo-DES pretreatment of sugarcane bagasse

Sr. No	pH	TDS	COD	BOD
1	2.9	700	2580	132
2	3	760	2620	134.5
3	3	780	2640	135.5
4	3	720	2660	137
5	3.1	740	2700	138.5

As required by the standard assay (APHA Standard Method 5210 B), the BOD_5_ was found to be 135.5 mg L^−1^ using a precise volumetric dilution fraction of (*p* = 0.0167) (5 mL/300 mL) after sample pre neutralization to a physiological baseline of pH 7.0 to protect the microbial seeding matrix. An extremely resistant pollution footprint is established by the baseline (BOD_5_\COD) ratio, which is computed at a very low 0.052. Two concurrent chemical processes are responsible for this extreme resistance to quick biological breakdown. First, accessible hemicellulose linkages are selectively broken by the low severity 1:5 treatment, but the waste stream is left with complex, high molecular weight dissolved polyphenolic lignin fractions that are resistant to quick microbial breakdown. Second, rather of taking part in solid mineral transitions, most of the prepared organic dicarboxylic acid stays dissolved in the liquid phase at this lower molar ratio. This raises the total COD value and lowers the waste stream's initial biological tractability index by leaving a high concentration of unreacted, chemically oxidizable organic matter in the liquid effluent.

With a steady pH of 3.5 and a raised average TDS value of 1060 mg L^−1^, the processing effluent produced by changing the treatment severity to the 1:10 asymmetric pseudo-DES configuration has a consistent, slightly modified physicochemical profile (Table 3). In these circumstances, the baseline COD, as determined by the requisite 5-fold predilution procedure necessary to satisfy the operating threshold (<800 mg L^−1^) of APHA Standard Method 5220 B, falls to 2000 mg L^−1^. Simultaneously, the average BOD_5_ shows an increasing trend to 161.5 mg L^−1^ after pre neutralization to pH 7.0, as determined using the precise volumetric dilution fraction of (*p* = 0.0167) as required by APHA Standard Method 5210 B. The biodegradability ratio (BOD_5_/COD) that results scale up to 0.081. The reactor's structural phase shifting mechanism is directly demonstrated by this noticeable decrease in liquid phase COD and rise in the biodegradability index. A large portion of the dicarboxylic acid is removed from the liquid phase when the oxalic acid level increases to a 1:10 ratio because it takes part in the *in situ* crystallization of insoluble calcium oxalate monohydrate onto the solid cellulose framework. The remaining chemically oxidizable load in the liquid effluent is naturally reduced by this migration of organic material into the solid biocomposite phase. The increased TDS concentration and the improved biological tractability of the wastewater stream in comparison to the lower 1:5 configuration is both explained by the increased proton density, which also speeds up the hydrolysis of resistant hemicellulose fractions into highly soluble, readily biodegradable monomeric sugars.

**Table 3:** Physicochemical characterization and pollution profile metrics of the processing effluent generated from the 1:10 asymmetric pseudo-DES pretreatment of sugarcane bagasse

Sr. No.	pH	TDS	COD	BOD
1	3.3	1040	1950	158
2	3.6	1100	1980	160.5
3	3.5	1060	2040	161.5
4	3.5	1080	2000	163
5	3.2	1020	2050	164.5

The processing effluent shows a steady, slightly acidic nature at the highest chemical severity (1:15 asymmetric pseudo-DES configuration), with a stable pH of 3.5 and a much higher average TDS value of 1500 mg L^−1^ (Table 4). Using the precise analytical decimal fraction of (*p* = 0.0167) under APHA Standard Method 5210 B, this optimized formulation yields an increased average BOD_5_ of 188.5 mg L^−1^ and a significantly lower average baseline COD of 1400 mg L^−1^ captured *via* the required 5-fold serial pre dilution required to conform to the working ceiling of APHA Standard Method 5220 B. The resulting (BOD_5_\COD) ratio reaches a maximum of 0.135, indicating a notable improvement in effluent biological tractability and biodegradability driven by improved hemicellulose sugar solubilization under the highly concentrated oxalic acid matrix. As the oxalic acid level increases from 1:5 to 1:15, a comparative analysis of the three asymmetric pseudo-DES ratios reveals extremely consistent and instructive environmental trends. The liquid phase pH easily stabilizes between 3.0 and 3.5, while the average TDS value nearly doubles from 740 mg L^−1^ to 1500 mg L^−1^, indicating advanced cell wall collapse. Surprisingly, the profile of organic pollution changes: baseline COD decreases from 2600 mg L^−1^ to 1400 mg L^−1^, whereas BOD_5_ increases from 135.5 mg L^−1^ to 188.5 mg L^−1^. While the concurrent release of highly mobile monomeric hemicellulose sugars structurally expands the liquor's overall biological tractability profile, the systematic removal of uncoordinated organic acid from the wastewater stream through solid phase mineral precipitation considerably reduces the residual chemical oxygen demand.^25,28^ The multiphase mass transfer taking place inside the reactor is directly responsible for this unusual, counterintuitive decrease in liquid phase COD. As it takes part in the *in situ* crystallization of insoluble calcium oxalate monohydrate (whewellite) onto the solid cellulose skeleton, a sizable amount of the organic dicarboxylic acid is gradually consumed and depleted from the wastewater stream at higher acid concentrations. Organic mass is moved from the liquid stream into the solid biocomposite matrix by this phase migration. Simultaneously, the biomass is selectively broken down by an intense proton donating network, resulting in an effluent with a higher percentage of easily fermentable, low molecular weight hemicellulose monomeric sugars and fewer complex, resistant aromatic lignin structures. This effectively changes the biodegradability index from a highly resistant 0.052 (at 1:5) to a much more manageable 0.135 (at 1:15). All three asymmetric pseudo-DES configurations consistently outperform traditional biomass processing methods from the standpoint of industrial sustainability. The total organic COD load produced by these organic acid formulations is still substantially smaller and far less harmful than the extremely corrosive, poisonous, and dangerous “black liquor” waste streams produced by industrial alkaline (NaOH) pulping. Furthermore, even at their highest concentrations, the dissolved solids from these organic, biocompatible blends are still much simpler to manage and neutralize in conventional wastewater facilities than the severe, extremely harmful inorganic sodium salt shocks typical of conventional industrial chemical pulping. The formation of persistent, bioaccumulative Adsorbable Organic Halogens (AOX), which are inevitably connected to traditional industrial chemical bleaching and extraction techniques, is entirely eliminated by this sustainable fractionation system because it only uses organic, biodegradable, and chlorine free components. This provides a highly responsible, environmentally friendly processing blueprint for contemporary biomass biorefining applications.

**Table 4:** Physicochemical characterization and pollution profile metrics of the processing effluent generated from the 1:15 asymmetric pseudo-DES pretreatment of sugarcane bagasse

Sr. No.	pH	TDS	COD	BOD
1	3.4	1480	1315	182
2	3.5	1450	1380	186
3	3.5	1520	1400	189
4	3.5	1500	1420	191
5	3.6	1550	1450	194.5

## 5 Conclusions

Using a ChCl:OA asymmetric Type 3 pseudo-DES system, this study presents a highly efficient and environmentally sustainable method for the multiphase fractionation of sugarcane bagasse, where processing performance is directly controlled by the HBD concentration. An ideal operating window that strikes a balance between selective cell wall delignification and the concurrent, *in situ* creation of a functional cellulose/calcium oxalate biocomposite matrix is established by changing the solvent composition to a highly asymmetric 1:15 molar ratio. This arrangement produces a remarkable 98% alkali insoluble structural component concentration and a 45% solid pulp recovery. Strong confirmation of the dual phase crystalline framework is obtained using various characterisation methods: SEM shows a clean, porous, stratified microarchitecture of thin, ribbon like fibrils seamlessly embedded with structured microcrystalline mineral domains; XRD confirms the retention of the native Cellulose I polymorph concurrently integrated with sharp, highly resolved Bragg reflections indexing to a crystalline whewellite phase; and FTIR tracks the complete attenuation of non-cellulosic lignin and hemicellulose bands along with mineral carbonyl sharpening at 1633.84 cm^−1^. This sophisticated biomass fractionation directly improves the liquor's downstream environmental characteristics. A large amount of the organic acid moves from the liquid stream into the solid composite phase as an insoluble mineral under the severe 1:15 arrangement, resulting in a unique phase migration. This phenomenon effectively raises the overall wastewater biodegradability ratio from a highly resistant 0.052 to a manageable 0.135 by systematically lowering the liquid phase baseline COD from 2600 mg L^−1^ to 1400 mg L^−1^ and raising the BOD_5_ from 135.5 mg L^−1^ to 188.5 mg L^−1^. This is in addition to the extensive hydrolysis of hemicellulose into soluble monomeric sugars. This chlorine free organic platform continues to have a significant environmental benefit over conventional chemical pulping and bleaching techniques across all evaluated molar ratios. This asymmetric pseudo-DES framework offers a clean, highly cohesive, and responsible approach for advanced, high value biomaterial fabrication within a circular green biorefinery model by maintaining a low, manageable TDS footprint (740 to 1500 mg L^−1^), removing dangerous inorganic sodium salt shocks, and totally avoiding toxic, bioaccumulative AOX generation.

## Conflicts of interest

There are no conflict of interest.

## Supplementary Material

RA-OLF-D6RA06342J-s001

## Data Availability

All data supporting the findings of this study, including the raw whole number titrimetric logs, multi-replicate environmental tracking sheets, and stoichiometric mass-balance parameters, are available within the article and supplementary information (SI) file. Supplementary information is available. See DOI: https://doi.org/10.1039/d6ra06342j.
