# Supplementary material for: Selective fractionation and in situ synthesis of cellulose/calcium oxalate biocomposites from sugarcane bagasse using asymmetric Type 3 deep eutectic solvents
Source: RSC Adv. 2026 Oct 22;16(48):65364–74. doi: 10.1039/d6ra06342j (PMC13689741; doi:10.1039/d6ra06342j)
Supplement: Supplementary file 1 [file RA-016-D6RA06342J-s001.pdf]

Supplementary Information (SI) for RSC Advances.
This journal is © The Royal Society of Chemistry

Supplementary Information

Selective Fractionation and In-Situ Synthesis of Cellulose/Calcium Oxalate Biocomposites from Sugarcane Bagasse Using Asymmetric Type 3 Deep Eutectic Solvents

Swapnil Deshmukh†, Prachi Tyagi†, Md. Arif Iqubal, Madhuri Kshirsagar, Y. M. Indi, S. R. Kamat and Shirishkumar Vhanbatte*

Department of Textiles, DKTE Society's Textile and Engineering Institute, Ichalkaranji (MH) India

This information document provides a comprehensive operational overview, rigorous thermodynamic phase characterization, and complete empirical transparency regarding the multi-phase sugarcane bagasse fractionation described in the main manuscript text. To secure absolute validation of our system and eliminate any technical or analytical ambiguities, this dossier compiles the explicit raw laboratory bench logs, titrimetric data profiles, and transport metrics governing both the green solvent complex and its corresponding liquid effluents. Section S1 outlines the core parameters verifying the solvent's unique supramolecular liquid phase bounds. While conventional stoichiometric deep eutectic solvents typically operate within balanced 1:1 or 1:2 molar boundaries, our highly asymmetric 1:15 ChCl:OA matrix functions as a coordinated asymmetric Type 3 pseudo-DES (or solvated eutectic fluid). Pure anhydrous oxalic acid possesses an exceptionally rigid, highly crystalline matrix with a characteristically high melting threshold of 189.5 °C due to dense inter-molecular self-association. Upon introducing choline chloride, the choline (Ch^+) and chloride (Cl^-) ions act as potent macroscopic lattice disruptors, permanently frustrating the long-range crystalline order of the bulk dicarboxylic acid. This structural disruption drives a massive 171.5°C thermodynamic melting point depression, enabling the highly concentrated 1:15 formulation to remain a perfectly clear, stable, non-precipitating, single-phase homogeneous liquid down to an ambient floor of 18.0 °C. Tracking these phase metrics validates the existence of an operational, deep eutectic hydrogen-bonding network without relying on mere literature extrapolations. Section S2 establishes the complete analytical audit trail for our downstream wastewater metrics in strict compliance with international guidelines (APHA Standard Method 5220 B). In chemical biorefining, monitoring COD is critical to map the cumulative

mass of chemically oxidizable matter and evaluate processing waste-stream compatibility. The raw, post-treatment liquid streams carry a highly concentrated organic load composed of dissolved hemicellulosic carbohydrates and degraded aromatic lignin fragments. Subjecting an undiluted 20.00 mL aliquot of this raw liquor to open reflux digestion would completely overwhelm the analytical system, leading to complete exhaustion of the primary chemical oxidant (10.00 mL of 0.25 N K₂Cr₂O₇). To resolve this threshold limitation, a systematic 5-fold serial pre-dilution (DF=5) was executed in the laboratory prior to digestion. This brought the raw oxidizable carbon components comfortably beneath the strict 800 mg/L operational working limit ceiling mandated by the open reflux protocol, protecting the chemical oxidant matrix and generating a fully verifiable titrimetric data footprint. The raw whole-number laboratory bench readings tracking exact FAS consumption volumes against a stable blank baseline are fully disclosed herein to verify the mathematical validity of our reported concentrations and provide an unassailable empirical audit trail.

Table S1. Thermodynamic Phase Profiles and Viscosity Shifts for the 1:15 Matrix

Thermodynamic & Transport Parameter	Experimental Value / Phase State	Physical Chemistry Significance & Context
Pure HBD Melting Threshold (T_m. raw)	189.5 °C	Rigid crystalline self-association floor of bulk anhydrous oxalic acid.
1:15 Pseudo-DES Freezing Point (T_f)	18.0 °C	Stable, clear, non-precipitating room-temperature fluid phase.
Net Thermodynamic Depression (ΔT_m)	171.5 °C	Direct thermodynamic proof of intensive long-range lattice frustration.
Macroscopic Phase / Atom Economy	Single-Phase Homogeneous Liquid	100% atom economy supramolecular blending with zero byproduct generation.
Initial Fluid Viscosity (Raw 1:15 Fluid Matrix)	~95 mPa·s	Dense, hyper-branched secondary and tertiary hydrogen-bonding molecular

		network.
Post-Dilution Viscosity (2:1 v/v H_2O Addition)	~12 mPa·s	High mobile mass transport achieved via 2:1 v/v H_2O addition; eliminates fiber entrapment.

S2. Step-by-Step Stoichiometric Derivation of the Calcium Incorporation Density

S 2.1. Theoretical Framework and Molecular Constants

To satisfy full scientific verification regarding the multi-phase composition of the sugarcane bagasse-derived biocomposites, the counter-intuitive 5% net mass accretion captured between the low-acidity baseline (1:5 ratio) and peak-severity processing run (1:15 ratio) was subjected to rigorous stoichiometric derivation. This gravimetric expansion represents the precise mass accumulation of highly dense, insoluble calcium oxalate monohydrate (CaC_2O_4. H_2O) crystals nucleated directly within the porous Cellulose I skeleton. The relative molecular and atomic masses used for this calculation are defined as follows:

- Atomic Mass of Calcium (MW_{Ca}): 40.08 g/mol
- Molecular Weight of Calcium Oxalate Monohydrate ($CaC_2O_4.H_2O$): 146.11 g/mol

The dimensionless stoichiometric mass fraction of elemental calcium securely localized within the mineral crystal lattice ($\square_{Ca}$) is calculated via Equation (S1):

$$\square_{Ca} = \frac{MW_{Ca}}{MW_{CaC_2O_4\square H_2O}} = \frac{40.08\ g/mol}{146.11\ g/mol} = 0.274314 \quad (S1)$$

S 2.2. Quantitative Substitution and Element Normalization

As documented in the main manuscript text and verified gravimetrically in Table 1, the initial dry mass of the raw agricultural bagasse feedstock (W1) used across all reactions is exactly 3.00 g. A net solid pulp yield expansion of 5% corresponds directly to an absolute mineral deposition weight

(ΔWmineral) of exactly 0.15 g (150.0 mg) localized within the biocomposite framework. Applying the derived stoichiometric mass fraction ($χ_{Ca}$), the absolute mass of the incorporated calcium cations (Ca^{2+}) is evaluated via Equation (S2):

$$\text{Mass of incorporated } Ca^{2+} = ΔW_{mineral} \times χ_{Ca} \quad \text{(S2)}$$

$$\text{Mass of incorporated } Ca^{2+} = 150.0 \text{ mg} \times 0.2743 = 41.145 \text{ mg of } Ca^{2+}$$

To eliminate processing scale dependencies and secure a standardized material attribute, this absolute elemental value is normalized directly against the initial dry weight of the raw agricultural biomass feedstock (W1 = 3.00 g), as defined in Equation (S3):

$$\text{Calcium Incorporation Density} = \frac{\text{Mass of incorporated } Ca^{2+} \text{ (mg)}}{W_1 \text{ (g)}} \quad \text{(S3)}$$

$$\text{Calcium Incorporation Density} = \frac{41.145 \text{ mg of } Ca^{2+}}{3.00 \text{ g of raw biomass}} = 13.715 \text{ mg/g}$$

Table S2: Summary of Gravimetric Variables and Stoichiometric Constants

Analytical Parameter	Symbol	Value	Units
Initial dry biomass feedstock	W1	3.00	g
Net gravimetric yield expansion	ΔY	5.00	%
Absolute synthesized mineral mass	$ΔW_{mineral}$	150.0	mg
Calcium lattice mass fraction	$□_{Ca}$	0.2743	Dimensionless
Absolute incorporated calcium	Ca^{2+}	41.145	mg
Ultimate chemical loading density	-	13.715	mg/g

Table S3. Experimental Parameters and Raw Data Logs for Effluent COD Evaluation (APHA Method 5220 B)

Operational	1:5 Molar Ratio	1:10 Molar	1:15 Molar	Protocol Significance &

Parameter (Units)	Matrix	Ratio Matrix	Ratio Matrix	Source
Analytical Standard Protocol	APHA 5220 B	APHA 5220 B	APHA 5220 B	Open Reflux Titrimetric Method
Primary Chemical Oxidant	$K_2Cr_2O_7$	$K_2Cr_2O_7$	$K_2Cr_2O_7$	10.00 mL of 0.25 N standard solution
Digestion Acid Matrix	H_2SO_4	H_2SO_4	H_2SO_4	30.00 mL concentrated solution with Ag_2SO_4 catalyst
Chloride Masking Agent	$HgSO_4$	$HgSO_4$	$HgSO_4$	Analytical crystalline pinch prior to refluxing
Thermal Digestion Windows	150°C for 2 h	150°C for 2 h	150°C for 2 h	Controlled standard reflux heating cycle
Back-Titration Indicator	Ferroin	Ferroin	Ferroin	2-3 drops (sharp green-blue to reddish-brown endpoint)
Titrant Normality (N)	0.1 N	0.1 N	0.1 N	Standardized Ferrous Ammonium Sulfate (FAS)
Analyzed Aliquot Volume (V)	20.00 mL	20.00 mL	20.00 mL	Volumetric capacity of the digested diluted sample
Blank FAS Titration Volume (A)	75.00 mL	75.00 mL	75.00 mL	Baseline distilled water control back-titration
Sample FAS Titration Volume (B)	62.10 mL	65.20 mL	68.00 mL	Raw whole-number laboratory bench readings
Pre-Digestion	5	5	5	Multi-fold water pre-dilution factor layout

Serial Dilution (DF)				
Direct Digestion Reading (mg/L)	516.00 mg/L	392.00 mg/L	280.00 mg/L	Measured baseline within protocol ceiling (<800 mg/L)
Final Calculated Baseline COD	2580.0 mg/L	1960.0 mg/L	1400.0 mg/L	Reported manuscript concentration matrix.

Mathematical Validation Formula and Phase Dynamics

The values compiled in Table S3 are derived in strict accordance with the core mathematical structure of APHA Method 5220 B (Equation 5):

$$COD_{(mg/L)} = \frac{(A\text{-}B)\times N\times 8000}{V} \times DF$$

- **1:5 Molar Ratio System:** [((75.00 mL - 62.10 mL) x 0.1 N x 8000) / 20.00 mL] x 5= 2580.0 mg/L
- **1:10 Molar Ratio System:** [((75.00 mL - 65.20 mL) x 0.1 N x 8000) / 20.00 mL] x 5= 1960.0 mg/L
- **1:15 Molar Ratio System:** [((75.00 mL - 68.00 mL) x 0.1 N x 8000) / 20.00 mL] x 5= 1,400.0 mg/L

As structurally demonstrated by these mathematical alignments, as the pseudo-DES molar severity expands, the remaining liquid-phase COD systematically declines. This is a direct consequence of the organic acid mass shifting out of the liquid waste stream and moving directly into the solid phase via the in-situ crystallization of insoluble calcium oxalate monohydrate (whewellite) onto the separating cellulose fiber frameworks, leaving behind a highly clean processing fluid characterized by maximized environmental tractability.

S4. Analytical Parameters and Replicate Logs for Effluent BOD₅ Evaluation (APHA Method 5210 B)

Methodological Controls and Protocol Compliance:

To guarantee absolute statistical rigor and eliminate any potential analytical ambiguities regarding downstream wastewater tractability, the complete raw laboratory biological replicates for the BOD_5

evaluations are compiled herein. Monitoring biological depletion parameters is vital to evaluate the true microbial tractability index (BOD_5/COD) of the processing streams as the pseudo-DES molar severity expands. In strict accordance with the regulatory mandates of APHA Standard Method 5210 B, the raw, highly acidic post-treatment effluents (baseline pH 3.0–3.5) were systematically neutralized to a stable, physiological baseline of 7.0±0.2 utilizing a dilute NaOH solution prior to inoculation. This critical step ensures a viable, non-toxic environment for the microbial seeding matrix, preventing cellular shock or premature termination of the biological decomposition cycle. Nutrient-enriched dilution water was prepared by blending 1.00 mL each of standard phosphate buffer, magnesium sulphate, ferric chloride, and calcium chloride solutions into 1.00 L of high-purity distilled water, followed by exhaustive aeration to achieve dissolved oxygen saturation. Neutralized sample aliquots of exactly 5.00 mL were introduced into standard 300 mL airtight glass incubation bottles and filled to capacity with the prepared seeded dilution water. This setup yields a precise, uniform sample dilution decimal fraction (p) of 0.0167 (5 mL/300 mL) across all evaluated streams.

- Analytical Standard Protocol: APHA 5210 B (5-Day Incubation Method)
- Airtight Incubation Capacity: 300 mL standard glass BOD bottles
- Sample Volumetric Aliquot: 5.00 mL (Neutralized to pH 7.0±0.2)
- Dilution Decimal Fraction (p): 0.0167 (5 mL/300 mL)
- Incubation Environment: 5 Days at exactly (20±1°C) in complete darkness
- Biological Seed Correction Factor (f): 0.95
- Initial Seed Control Blank DO (B1): 8.15 mg/L
- Final 5-Day Seed Control Blank DO (B2): 7.73 mg/L

Table S4: Raw Dissolved Oxygen Titration Logs

Processing Ratio	Initial Sample DO (D1)	Final 5-Day DO (D2)	Net DO Depletion (D1 –D2)	Standard Equation 8 Evaluation

1:5 molar ratio matrix	7.95 mg/L	5.35 mg/L	2.60 mg/L	$\frac{2.600 - 0.399}{0.0167}$ = 131.796
1:10 molar ratio matrix	7.95 mg/L	4.91 mg/L	3.04 mg/L	$\frac{3.040 - 0.399}{0.0167}$ = 158.143
1:15 molar ratio matrix	7.95 mg/L	4.51 mg/L	3.44 mg/L	$\frac{3.440 - 0.399}{0.0167}$ = 182.095
